# Removal of Rifampicin and Rifaximin Antibiotics on PET Fibers: Optimization, Modeling, and Mechanism Insight

**DOI:** 10.3390/polym17152089

**Published:** 2025-07-30

**Authors:** Elena Fasniuc-Pereu, Elena Niculina Drăgoi, Dumitru Bulgariu, Maria-Cristina Popescu, Laura Bulgariu

**Affiliations:** 1 Department of Environmental Engineering and Management, “Cristofor Simionescu” Faculty of Chemical Engineering and Environmental Protection, Gheorghe Asachi Technical University of Iaşi, 700050 Iaşi, Romania; elena.fasniuc@gmail.com; 2 Department of Chemical Engineering, “Cristofor Simionescu” Faculty of Chemical Engineering and Environmental Protection, Gheorghe Asachi Technical University of Iaşi, 700050 Iaşi, Romania; elena-niculina.dragoi@academic.tuiasi.ro; 3 Department of Geology, Faculty of Geography and Geeology, Al.I.Cuza University of Iaşi, 700506 Iaşi, Romania; dbulgariu@yahoo.com; 4 Branch of Geography, Filial of Iaşi, Romanian Academy, 700506 Iaşi, Romania; 5 Department of Physical Chemistry of Polymers, Petru Poni Institute of Macromolecular Chemistry of the Romanian Academy, 700506 Iaşi, Romania; cpopescu@icmpp.ro

**Keywords:** adsorption, antibiotics, polyethylene terephthalate fibers, artificial neural networks, modeling, mechanism

## Abstract

The removal of antibiotics from aqueous media along with their recovery is still an open research topic, due to their practical and economical importance. Adsorption allows these two objectives to be achieved, provided that the adsorbent used is chemically and mechanically stable and has a low preparation cost. In this study, PET (polyethylene terephthalate) fibers, obtained by mechanically processing PET waste, were used for the adsorption of rifampicin (RIF) and rifaximin (RIX) antibiotics from aqueous media. The experimental adsorption capacity of PET fibers for the two antibiotics (RIF and RIX) was determined at different pH values (2.0–6.5), adsorbent dose (0.4–20.0 g/L), contact time (5–1440 min), initial antibiotic concentration (4.0–67.0 mg/L), and temperature (10, 22, and 50 °C); the experimental values of these parameters were analyzed using a neuro-evolutive technique (ANE) combining sequential deep learning (DL) models with a differential evolution algorithm. The obtained optimal ANN-DL algorithm was then used to obtain the optimal models for the adsorption of RIF and RIX on PET fibers, which should adequately describe the adsorption dynamics for both antibiotics. The adsorption processes are spontaneous and endothermic (ΔG < 0, ΔH > 0) and are described by the Langmuir model (R^2^ > 0.97) and the pseudo-second order kinetic model (R^2^ > 0.99). The retention of RIF and RIX on the surface of PET fibers occurs through physicochemical interactions, and the FTIR spectra and microscopic images support this hypothesis. The presence of inorganic anions in the aqueous solution leads to an increase in the adsorption capacities of RIF (max. 7.6 mg/g) and RIX (max. 3.6 mg/g) on PET fibers, which is mainly due to the ordering of water molecules in the solution. The experimental results presented in this study allowed for the development of the adsorption mechanism of RIF and RIX on PET fibers, highlighting the potential practical applications of these adsorption processes.

## 1. Introduction

Antibiotics continue to have a significant impact on modern societies, both by improving public health and by enhancing the quality of water, food, and overall hygiene standards [1,2]. Numerous types of antibiotics (semi-synthetic or synthetic) are currently used to treat a wide range of pathologies (both in humans and animals) due to their effectiveness in stopping the development of infections [3,4]. Among these, rifampicin (RIF) is a major and effective drug for the treatment of tuberculosis [5,6], while rifaximin (RIX) is a broad-spectrum antibiotic used to prevent recurrent hepatic encephalopathy [7,8].

But the excessive and improper use of antibiotics leads to their accumulation in wastewater (domestic or industrial), which, if not properly treated, contaminates water sources [9,10,11]. The increase in antibiotic concentrations in water sources leads to a rise in bacterial resistance [12], and has particularly serious environmental consequences, as it affects both the quality of ecosystems where these water sources are found and human health [13,14]. Studies in the literature show that the misuse of antibiotics is one of the top 10 threats to global public health faced by societies today [15,16]. For this reason, finding an appropriate method for the removal of antibiotics from aqueous media, which is both technologically and economically feasible, remains an open research direction for which pertinent solutions are being sought.

So far, a wide range of methods have been used for the removal of antibiotics from aqueous environments, including photocatalytic degradation [17,18], advanced oxidation [19,20], biodegradation [21], membrane filtration [22], and adsorption [23,24]. All these methods have proven to be effective under certain operating conditions and have the potential to be adapted for large-scale applications. Compared to the other mentioned methods, adsorption has the advantage of allowing both the efficient removal of antibiotic molecules from aqueous environments and their subsequent recovery, without the risk of molecular degradation [24]. Additionally, the low cost and ease of operation under various experimental conditions are two other important advantages of adsorption processes [24,25].

Numerous inorganic or organic materials have been reported in the literature as adsorbents for the retention of various antibiotics from aqueous media [26,27,28]. Among these, PET fibers may represent a viable alternative, as they have high chemical and thermal stability [29,30], can be used in a large number of adsorption/desorption cycles, are easy to procure, and have a low cost [30]. Moreover, since PET fibers can be obtained by recycling PET waste [29], their use as an adsorbent may contribute to reducing the environmental contamination caused by such waste, in accordance with the principles of circular economy. Based on these considerations, PET fibers were selected as the adsorbent in this study and tested for the removal of rifampicin (RIF) and rifaximin (RIX) from aqueous media.

Over the past decade, machine learning (ML) and, particularly, deep learning (DL), has seen tremendous developments and has found applications in several fields. For water and wastewater treatment, examples of ML/DL strategies successfully applied include the following: (i) the use of a neural network model to predict the adsorption capacity of certain composites in the adsorption processes of specific antibiotics [31], (ii) the prediction of the efficiency of antibiotic removal based on the Nonlinear Least Squares Support Vector Machine (N-LSSVM), Generalized Regression Neural Network (GRNN), and Response Surface Methodology (RSM) [32], or (iii) determining the evolution of adsorption processes within specific ranges of variation in experimental parameters [33]. In this study, a sequential deep learning (DL) model was used to analyze the adsorption of rifampicin (RIF) and rifaximin (RIX) from aqueous media on PET fibers. The optimization was performed through the differential evolution algorithm (DE).

The differential evolution algorithm (DE) is a simple, easy-to-implement strategy that has proved effective (in its simple or modified form) in solving many problems in multiple areas [34]. Once the optimal model was established, it was then used for process optimization, where, based on the same DE strategy, the optimal conditions were determined to achieve maximum efficiency in the removal of the two antibiotics through adsorption onto PET fibers. Although the tendency in the literature is to use multiple optimization strategies for each particular case (parameter and process optimization) [35], this study focuses on a simplified approach using the same optimizer. This simplifies the overall modeling and optimization procedure and reduces its complexity (both from the implementation perspective and in terms of setting the optimizer parameters).

Therefore, the main objective of this study is to provide a more comprehensive and well-argued description of the adsorption processes of RIF and RIX on PET fibers, in order to highlight their usefulness in practical applications. For this reason, the results obtained in establishing the optimal conditions and modeling the adsorption processes are discussed in detail in this study, and they serve as the starting point for developing the adsorption mechanism of RIF and RIX on PET fibers.

## 2. Materials and Methods

### 2.1. Materials and Reagents

PET fibers (6.7 × 64 SD), purchased from a local company (GreenFiber International Company, Iaşi, Romania), were obtained via the hot mechanical processing of PET waste. To be used in laboratory studies, the PET fibers were cut to a length of 0.5–1.0 cm, washed with a 0.1 mol/L HNO_3_ solution to remove impurities, and then rinsed with distilled water until reaching a neutral pH (pH > 6.5). The washed PET fibers were air-dried at room temperature (22 ± 2 °C) and stored in desiccators.

Rifampicin (RIF) and rifaximin (RIX) (monomer, purity > 99.9%) were purchased from Sigma Aldrich and used without further purification. Some important chemical characteristics of these two antibiotics are summarized in Table 1.

The stock solutions of the two antibiotics (240 mg/L) were obtained by dissolving an appropriate amount of solid antibiotic in ethanol (96%, Chemical Company, Iași, Romania). The stock solutions were prepared before each set of experiments and were not stored in the refrigerator for more than 2–3 days. The working solutions were prepared from the stock solutions by dilution with distilled water. A 1 mol/L HNO_3_ solution was used to adjust the pH of the working solutions.

### 2.2. Adsorption Experiments

All adsorption experiments were conducted in batch systems, varying one factor at a time. pH (2.0–6.5), adsorbent dose (0.4–20.0 g/L), contact time (5–1440 min), initial antibiotic concentration (4.0–67.0 mg/L), and temperature (10, 22, and 50 °C), to determine the optimal conditions for the adsorption of RIF and RIX on PET fibers. In each case, 100 mL Erlenmeyer flasks were used, into which the desired amount of PET fibers and 25 mL of antibiotic solution of the given concentration were added. At the end of the experiments, the PET fibers were removed from the solution, and the residual concentration of the two antibiotics was analyzed spectrophotometrically (Carry 60 UV-VIS spectrophotometer (Agilent, Santa Clara, CA, USA), 1 cm glass cell, phosphate buffer (pH = 7.0), RIF: λ = 470 nm, RIX: λ = 444 nm, against distilled water). The quantitative evaluation of the adsorption processes was carried out using the adsorption capacity (q, mg/g) and the removal percent (R, %), calculated from the following equations:

(1) $$q=\frac{(c_{0}-c)\cdot V}{m}$$

(2) $$R=\frac{c_{0}-c}{c_{0}}\cdot 100$$

where *c_o_* is the initial concentration of antibiotics, (mg/L); *c* is the equilibrium concentration of antibiotics, (mg/L); *m* is the mass of adsorbent, (g); and *V* is the volume of solution, (L).

### 2.3. Characterization of PET Fibers

The presence of functional groups on the surface of PET fibers was highlighted using FTIR spectra, recorded before and after the retention of the two antibiotics (Bio-Rad Spectrometer (Berlin, Germany), KBr pellet technique, spectral range = 400–4000 cm^−1^, resolution = 4 cm^−1^). The surface morphology of PET fibers, before and after antibiotic adsorption, was analyzed using SEM microscopy (SEM Hitachi S3000N (Tokyo, Japan), 20 kV) and optical microscopy (Optika Jena, Berlin, Germany, digital 2200X) at different orders of magnitude.

### 2.4. ANN-DE Stategy

**Figure 1:**
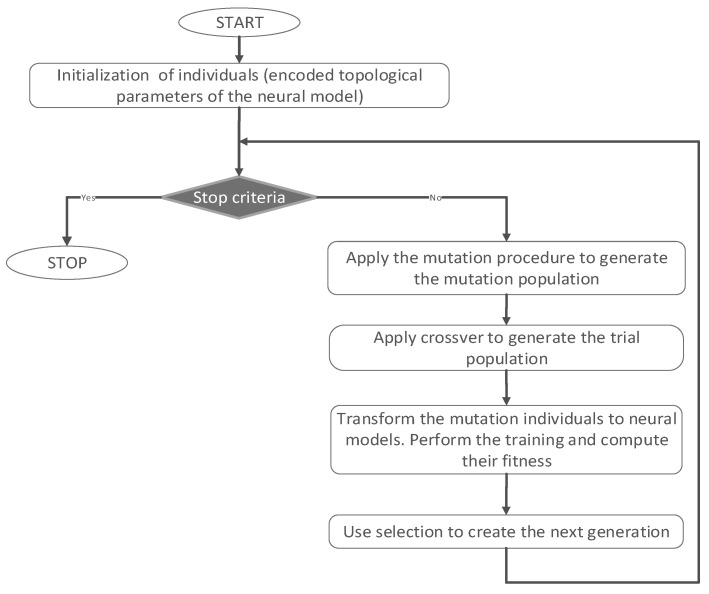
ANN-DE strategy used for optimization of experimental data.

In this study, the adsorption of RIF and RIX antibiotics on PET fibers is determined based on a series of process parameters that include antibiotic type, pH, adsorbent dose (g/L), contact time (min), antibiotic concentration (mg/L), and temperature (°C). This is performed using a modeling strategy based on a combination of artificial neural networks (ANNs) and DE algorithms. While most process parameters are (or can be considered) continuous features, the antibiotic type (RIF or RIX) is categorical. As such, one hot encoding strategy is applied. In this hot encoding strategy, each unique categorical value is represented as a new feature, with values 0 and 1 indicating its presence. Consequently, the total number of features considered in the modeling phase is seven, and the number of outputs is one. If the characteristics of the process determine the number of inputs and outputs considered in the modeling phase, the rest of the topological parameters for the ANN (number of hidden layers and neurons in each hidden layer) can be manually set, and their optimal values are problem-dependent. Therefore, this study uses DE to perform automatic topological optimization. DE is a simple and practical approach inspired by the Darwinian principle of evolution. It evolves a series of potential solutions (initially randomly generated) through a series of steps like mutation, crossover, and selection. The simplified illustration of the ANN-DE strategy is presented in Figure 1. As observed from the first step in Figure 1, since DE employs vectors formed from real values, an encoding strategy is necessary to transform the considered ANN parameters into structures DE can work with.

In this study, direct encoding is applied to perform this transformation. After the mutation and crossover steps, the decoding procedure is applied to transform the DE individual representing the ANN topology into an actual model that can be further trained with Adam (Adaptive Moment Estimation) [39]; its performance is included in the fitness function used to determine the best solutions. The DE variant used is DE/current-to-best/bin, which implies that (i) in the mutation phase, the based individual is represented by the current individual, and the differential terms include the best so far solution; (ii) the crossover type is binomial. The parameters F and Cr control the crossover and mutation phases and, in this case, a simple, self-adaptive procedure is applied that includes them in the individual and develops as such. More details about the DE optimization strategy and its combination with ANNs can be found in reference [35].

After determining the best model, the DE algorithm is applied for process optimization. The same variant and control parameters as in the case of model identification are applied. The difference between the model and process optimization consists of the parameters encoded into the DE vectors. In the case of model optimization, the DE vector contains the topological ANN parameters.

**Figure 2:**
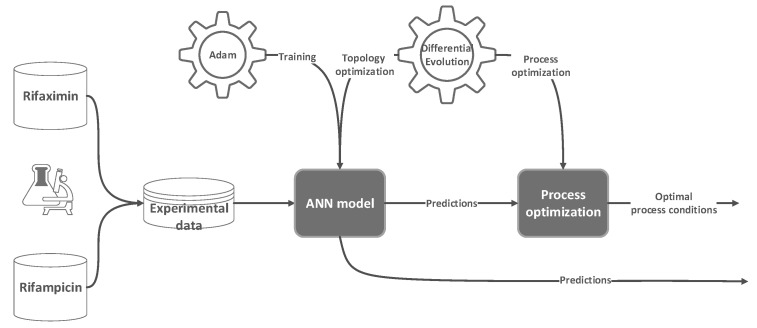
Schematic illustration of modeling and optimization algorithm.

In contrast, for process optimization, the DE vector contains the process parameters analyzed in the experimental phase and then considered as features for the ANN model. Another difference between model and process optimization is represented by how the fitness function is computed. In the model optimization phase, it is based on the loss function of the ANN from the training phase. In contrast, in the process optimization phase, it is based on the predictions generated by the ANN using the specific combination of process parameters. The simplified schema of the overall modeling and optimization algorithm is presented in Figure 2. The implementation is performed in Python 3.10 using Tensorflow, Keras, and SHAP.

## 3. Results and Discussion

### 3.1. Experimental Characteristics of Adsorption Processes

**Figure 3:**
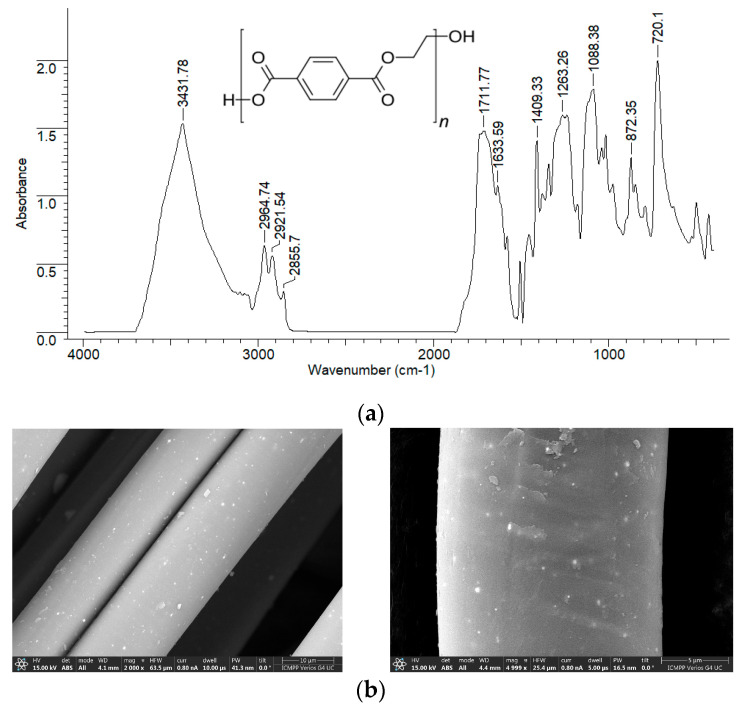
FTIR spectrum (**a**) and SEM images (**b**) of PET fibers.

Due to the way they are obtained (mechanical processing of PET waste), PET fibers are expected to have a relatively smooth surface and a reduced number of superficial functional groups. These structural particularities are clearly highlighted by the FTIR spectrum and the SEM images, recorded experimentally (Figure 3). However, the presence of hydroxyl groups (inter molecular hydrogen bonding) (3432 cm^−1^), carbonyl groups (1711–1633 cm^−1^), C-H bonds in saturated hydrocarbon radicals (2964–2855 cm^−1^), C-O-C bonds (1088 cm^−1^ cm), and the aromatic nucleus (872–720 cm^−1^) can be easily observed in the FTIR spectrum due to the high intensity of the absorption bands (Figure 3a).

Moreover, although the surface of PET fibers is smooth, increasing the magnification of SEM images reveals a series of irregularities (Figure 3b) that may be important for the adsorption processes. All these structural features make PET fibers suitable as adsorbents for retaining large organic molecules from aqueous media.

**Figure 4:**
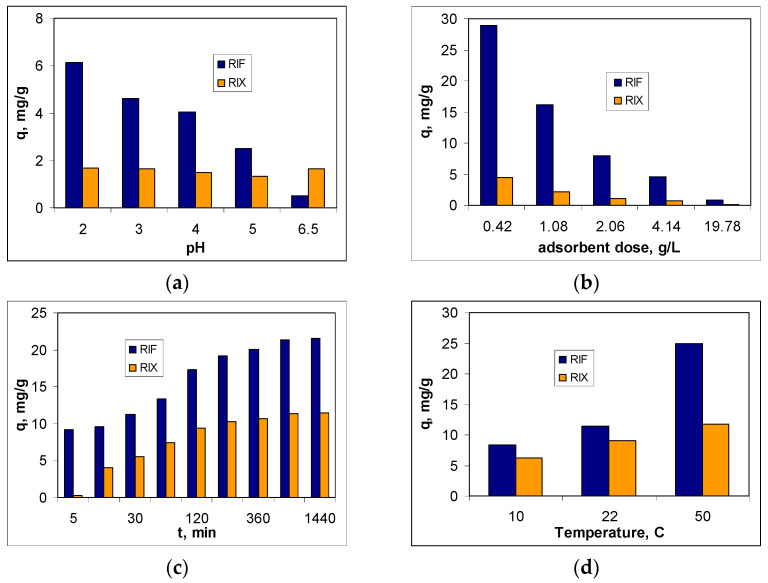
Effect of initial solution pH (**a**), adsorbent dose (**b**), contact time (**c**), and temperature (**d**) on adsorption of RIF and RIX onto PET fibers.

The next step in evaluating the feasibility of using PET fibers for the removal of RIF and RIX from aqueous environments is to examine the influence that experimental parameters (pH, adsorbent dose, contact time, and temperature) have on the efficiency of adsorption processes. The influence of each of these parameters on the adsorption capacity of PET fibers for the two studied antibiotics (RIF and RIX) is illustrated in Figure 4.

The influence of pH on the efficiency of RIF and RIX retention on PET fibers was studied in the pH range between 2.0 and 6.5, and the adsorption capacity values are presented in Figure 4a. The selection of this pH range was made considering the acidity constant values reported in the literature for RIF (1.8 and 7.9) [36] and RIX (2.08, 3.02, 7.28, 9.32, and 12.55) [37], and the other experimental conditions were adsorbent dose of 2.0 g/L, initial concentration of 21.1 mg/L, contact time of 3 h, and temperature of 22 °C. The obtained results show that at pH 2.0, the highest amount of RIF is retained (56.60%), and further increasing the pH leads to a decrease in the adsorption efficiency of this antibiotic. In the case of RIX, the highest values of q are obtained at both pH = 2.0 (1.68 mg/g) and pH = 6.5 (1.65 mg/g), although the variation in adsorption capacity across the entire pH range is quite small (0.30 mg/g).

It is well known that the variation in adsorption capacity with pH provides clear evidence that electrostatic interactions are involved in the adsorption process [23]. Therefore, the ionic form of the antibiotics and the functional groups of PET fibers play an important role in facilitating such interactions. At pH 2.0 (strong acid media), RIF and RIX molecules are negatively charged (due to the ionization of the first functional group), while the carboxyl/carbonyl groups of PET fibers are undissociated/partially protonated (pKa1 for terephtalic acid (monomer) is 3.54 [40]). Thus electrostatic interactions (rather weak) occur between the antibiotic molecules (RIF and RIX) and the functional groups on the PET fibers’ surface, interactions facilitated by the hydrogen ions (of marginal carboxyl groups or protonated carbonyl groups). Increasing the pH of the aqueous solution, although leading to an increase in the ionization degree of the antibiotic molecules, significantly reduces the formation of positive charges on the surface of the PET fibers. Consequently, the adsorption capacity decreases with increasing pH, and this is especially evident in the case of RIF adsorption. In the case of RIX molecules, increasing pH has a smaller influence on the efficiency of the adsorption process (Figure 4a). This is because (i) the experimental values of the adsorption capacities are small, and the modification of the values at the first decimal place does not represent a significant variation, and (ii) unlike RIF (which has a single functional group dissociable in acidic media), RIX has two functional groups that can dissociate in acid media (pKa_1_ = 2.08 and pKa_2_ = 3.02), which leads to an increase in the number of negative charges that can be involved in electrostatic interactions. Based on these observations, the optimal pH for the adsorption of RIF and RIX on PET fibers was considered to be 2.0, and this value was used in all subsequent experiments.

The effect of adsorbent dose on the adsorption efficiency of RIF and RIX was examined in the range of 0.4–20 g/L, while the values of the other experimental parameters (pH = 2.0, initial concentration = 21.1 mg/L, contact time = 3 h, temperature = 22 ± 1 °C) were kept constant. As can be seen in Figure 4b, as the amount of PET fibers increases, the adsorption capacity values (q, mg/g) decrease for both antibiotics (RIF and RIX). On the other hand, increasing the adsorbent dose within this range leads to an improvement in the removal percentage from 58.9 to 76.4% for RIF and from 8.9 to 12.7% for RIX (). This opposite variation in the parameters q and R with the increase in the amount of PET fibers is a consequence of the variation in the ratio between the number of active centers of the adsorbent and the number of antibiotic molecules in the aqueous solution. Increasing the adsorbent dose causes the antibiotic molecules present in the solution to be retained on an increasing amount of PET fibers, which means that the ratio between the number of antibiotic molecules and the number of active centers of the adsorbent will decrease. Under these conditions, considering the definition relationships (see Equations (1) and (2)), the adsorption capacity values decrease, while the removal percentage values increase. However, the decrease in q is not proportional to the increase in R. Thus, while the adsorption capacity values decrease by more than 36 times in the case of RIF and by more than 33 times in the case of RIX (Figure 4b), the R values (%) increase by only 17% in the case of RIF and by approximately 4% in the case of RIX. Under these conditions, an adsorbent dose of 0.4 g/L can be considered optimal and was used for subsequent experiments.

The influence of contact time on the adsorption efficiency of RIF and RIX on PET fibers was examined over a fairly wide range (5–1440 min), under the following experimental conditions: pH = 2.0, adsorbent dose = 0.4 g/L, initial concentration = 21.1 mg/L, temperature = 22 ± 1 °C. The adsorption capacity values obtained for the adsorption of RIF and RIX on PET fibers are illustrated in Figure 4c. According to experimental dependencies, the amount of RIF and RIX retained on PET fibers increases with contact time in the range of 5–180 min, after which it remains practically constant (320–1440 min). This variation in q as a function of contact time is valid for both antibiotics (RIF and RIX), although the adsorption capacity values differ significantly. This behavior suggests that, in the studied adsorption processes, the availability of RIF and RIX molecules to interact with the active centers of the adsorbent plays an important role. Initially, when most functional groups on the surface of PET fibers are free, the retention of RIF and RIX occurs fairly quickly. After the functional groups of PET fibers are occupied by antibiotic molecules, the adsorption process rate decreases significantly. The calculated adsorption capacity values remain practically constant, suggesting that equilibrium has been reached. Thus, for a contact time of 180 min, the retention percentage is 37.5% for RIF and 20.5% for RIX, while increasing the contact time to 1440 min leads to only a 6% increase for RIF and a 2.5% increase for RIX. Therefore, a contact time of 180 min can be considered sufficient for the studied adsorption processes to reach equilibrium.

Figure 4d shows the effect of temperature on the adsorption efficiency of RIF and RIX on PET fibers. The other experimental parameters were maintained at the previously established optimal values (pH = 2.0, initial concentration = 22.1 mg/L, adsorbent dose = 0.4 g/L, contact time = 180 min), while the temperature was adjusted at 10, 22, and 50 °C. It should be noted that both antibiotics (RIF and RIX) are thermally stable up to temperatures above 200 °C [36,37], and therefore, there is no problem with their decomposition during adsorption processes.

As can be seen from Figure 4d, increasing temperature leads to an increase in adsorption capacities for both RIF and RIX, although this increase depends on the nature of the antibiotic in the aqueous solution. This, if in the case of RIF, the adsorption capacity increases by more than 3 times (from 8.30 to 24.91 mg/g) with the increase in temperature from 10 to 50 °C, in the case of RIX, the increase in adsorption capacity is more modest (1.8 times, from 6.24 to 11.73 mg/g) under the same experimental conditions. This variation in q values with increasing temperature suggests the endothermic nature of RIF and RIX adsorption on PET fibers and is most likely determined by the increased mobility of antibiotic molecules, which facilitates their retention on the adsorbent surface. However, increasing the temperature from 22 °C (ambient temperature) to 50 °C improves the removal percent values by only 27.12% in the case of RIF and by 3.74% in the case of RIX, which does not justify the energy consumption required to maintain the adsorption systems at a temperature of 50 °C. Therefore the temperature of 22 °C was considered to be much more suitable for the adsorption of RIF and RIX on PET fibers, both in terms of the efficiency of the adsorption process and from an economical point of view, and this value was selected as optimal.

### 3.2. Optimization of Adsorption Processes

**Figure 5:**
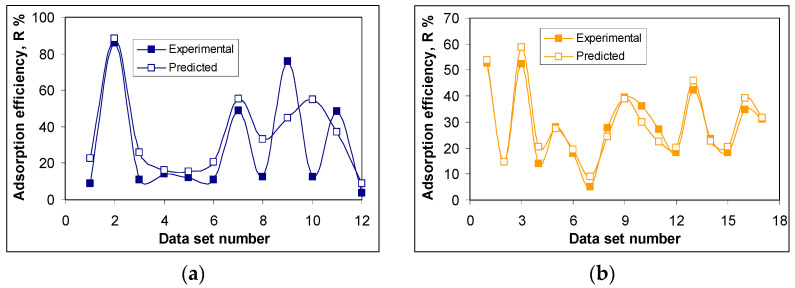
Comparison between experimental and ANN-predicted data for adsorption of RIF (**a**) and RIX (**b**) onto PET fibers.

One of the main objectives of this study was to establish the experimental conditions for which the adsorption efficiency of the two antibiotics (RIF and RIX) on PET fibers is the highest possible. To achieve this objective, an optimization study was conducted. After collecting the experimental data, the obtained data set (comprising a total of 97 experiments) was normalized using the min–max approach [35], and the data was randomly distributed into training and testing phases (70% for training and 30% for testing). Next, the DE algorithm was applied to determine the optimal model. The type of neural model considered in this study is the sequential model with dense layers. Since the DE variant used in this study uses fixed-length vectors due to the applied direct coding strategy, a limitation on the number of hidden layers and number of neurons in each hidden layer must be imposed. Therefore, a topology with a maximum of five hidden layers and 20 neurons in each hidden layer was considered. For the neurons in the hidden layers, the activation function is ReLU, and for the output neurons, it is set to linear. A L1 kernel regularization rate of 0.01 is considered, and for the Adam optimizer, a fixed learning rate of 0.05 is set based on a series of preliminary runs. To avoid over-training, the validation split in the training phase was set to 0.2. For the DE algorithm, the total number of generations was set to 30, and due to its stochastic nature, the optimal model was selected as the best one from 10 runs. The best model obtained has a topology with seven input features, four hidden layers with 19, 5, 6, and 3 neurons, respectively, and one output, corresponding to the adsorption efficiency (R, %). Table 2 presents the statistical indicators that measure the performance of this model, and Figure 5 compares the predicted and the experimental values for the training sub-set.

As can be seen from Figure 5, there is very good agreement between the values predicted by the ANN algorithm and the experimental ones, in terms of the adsorption efficiency (R, %), for both antibiotics (RIF and RIX). The highest values of the removal percent are obtained for the conditions of pH = 2.0, adsorbent dose = 0.4 ± 0.04 g/L, contact time = 180 min and temperature = 22 ± 1 °C. For an initial concentration of 21.1 mg RIF/L and 23.4 mg RIX/L, respectively, the removal percent calculated using the ANN algorithm is 88.13% (compared to 85.94%, experimental) for RIF, and 58.78% (compared to 52.42%, experimental) for RIX, respectively.

Therefore, these experimental conditions (pH = 2.0, adsorbent dose = 0.4 g/L, contact time = 180 min, and temperature = 22 ± 1 °C) allow for efficient adsorption of RIF and RIX onto PET fibers, at least in the range of low initial concentrations (below 25 mg/L).

**Figure 6:**
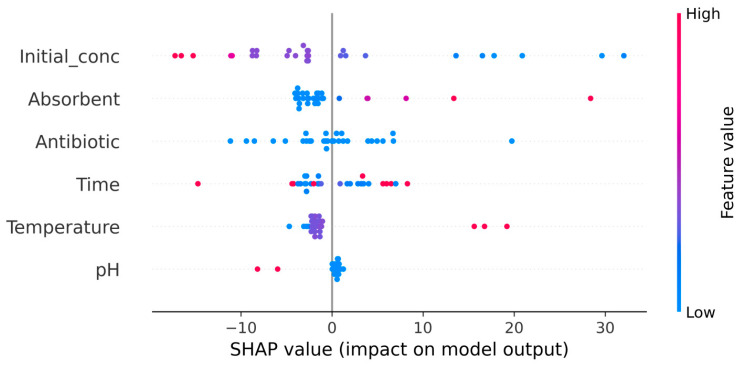
Variation in the SHAP values for the adsorption of RIF and RIX onto PET fibers.

However, there are also certain differences between the values predicted by the ANN algorithm and those obtained experimentally. The most notable are those obtained in the case of data sets number 9 and 10 for RIF adsorption (Figure 5a). These data sets correspond to RIF adsorption at pH = 2.0, adsorbent dose = 19.9 g/L, contact time = 180 min, temperature = 22 °C (data set 9), and at pH = 2.0, adsorbent dose = 0.42 g/L, contact time = 1440 min, temperature = 22 °C (data set 10);the difference between the predicted R, % values, and those obtained experimentally is greater than 30% (+31.06% for data set 9, and −42.20% for data set 10). These differences may be caused by (i) the limitations of the ANN algorithm in predicting R% values—both cases (data sets 9 and 10) correspond to extreme limits of the experimental range, and the ANN model was unable to predict them efficiently—or (ii) the different contributions of each parameter (pH, adsorbent dose, contact time, temperature) on the performance of adsorption processes. If the first cause is quite unlikely (but not impossible), to check the second possible cause, the SHapley Additive exPlanations (SHAP) values [39] were computed using the SHAP module. These values provide an objective way to explain how each parameter contributes to the adsorption processes, and the representation of SHAP values for the test data is shown in Figure 6.

The parameters on the Y-axis are ordered by their importance, while the SHAP values are represented on the X-axis. Positive SHAP values indicate a positive trend (an increase in these values leads to an increase in adsorption efficiency), whereas negative SHAP values indicate a negative trend (an increase in these values leads to a decrease in adsorption efficiency). The distance of SHAP values from 0 reflects the impact that each parameter has on influencing the efficiency of adsorption processes.

The SHAP values illustrated in Figure 6 show that the initial concentration of the two antibiotics has the greatest contribution to achieving RIF and RIX adsorption on PET fibers, while pH has the smallest contribution. In addition, high initial antibiotic concentrations (indicated by red dots) lead to low adsorption efficiency (which decreases as the initial concentration increases). Increasing the adsorbent dose, contact time, and temperature can lead to improved adsorption process efficiency (Figure 6), but economic considerations are also taken into account when determining the experimental values of these parameters. On the other hand, increasing the pH negatively affects the efficiency of adsorption processes, most likely due to changes in the dissociation state of antibiotic molecules (Figure 6). In turn, the nature of the antibiotic influences the efficiency of the adsorption process. The blue color of the dots (Figure 6) is due to the fact that only two antibiotics (RIF and RIX) were used in this study, but the distribution of these dots confirms the different efficiencies of RIF and RIX, observed experimentally.

### 3.3. Isotherm and Kinetic Modeling of Adsorption Processes

For the quantitative evaluation of RIF and RIX adsorption on PET fibers, it is necessary to model the adsorption isotherms and the kinetic curves obtained experimentally, under the conditions established as optimal. In this study, the adsorption isotherms were analyzed using the Langmuir, Freundlich, and Temkin models, while for the kinetic curves, the pseudo-first order, pseudo-second order, and intra-particle diffusion models were used.

**Figure 7:**
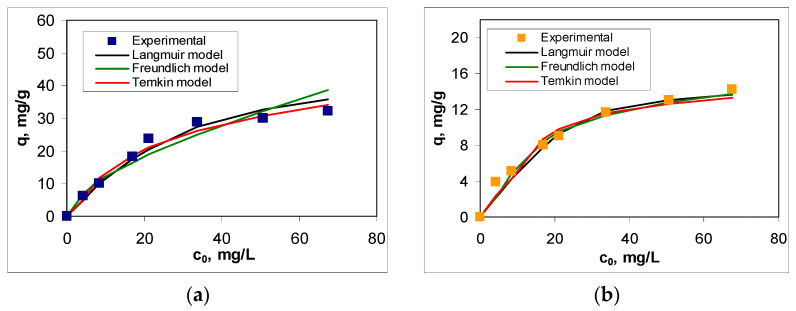
Experimental and calculated isotherms for adsorption of RIF (**a**) and RIX (**b**) onto PET fibers (experimental conditions: pH = 2.0, adsorbent dose = 0.4 g/L, contact time = 180 min, and temperature = 22 ± 1 °C).

The experimental isotherms (Figure 7) were obtained by varying the initial concentration of RIF and RIX within the range of 4.22–67.5 mg/L, while the other parameters were kept constant (at optimal values). As expected, increasing the initial antibiotic concentration leads to an increase in adsorption capacity over the entire concentration range. However, a detailed analysis of the obtained isotherms shows that (i) they are nonlinear—the increase in adsorption capacity with increasing initial concentration is more pronounced in the low concentration range (up to 25 mg/L) than in the high concentration range (above 25 mg/L)—and (ii) the efficiency of adsorption processes significantly depends on the nature of the antibiotic in the aqueous solution—the adsorption capacities obtained for RIF are more than 2 times higher than those obtained for RIX (Figure 7).

These particularities suggest that the surface of PET fibers contains a limited number of active sites (functional groups) that can participate in adsorption processes (as evidenced by the FTIR spectrum—see Figure 3). Moreover, the retention of antibiotic molecules involves specific interactions in which their chemical structure plays a crucial role. In other words, the ability of RIF and RIX molecules to interact with the functional groups of PET fibers represents the driving force behind the adsorption process. Once these functional groups on the adsorbent surface are occupied (through the binding of RIF and RIX molecules), an increase in the initial concentration leads to a much smaller variation in adsorption capacity (Figure 7), suggesting that saturation has been reached. It should be noted that, according to experimental data (Figure 7), the saturation of PET fibers can be observed at concentrations higher than 25 mg/L (for both studied antibiotics), highlighting the practical applicability of this adsorbent.

The selection of Langmuir, Freundlich, and Temkin models [41] for the analysis of experimental data allows the determination of how the adsorption of RIF and RIX occurs on PET fibers (mono- or multilayer), as well as the predominant type of interactions responsible for binding antibiotic molecules to the adsorbent surface. The isotherms obtained for each model are also illustrated in Figure 7, and the characteristic parameters of these models are summarized in Table 3. The selection of the most suitable model was made using statistical parameters (R^2^, RMSD, and Chi-square).

As can be seen from Table 3, the values of statistical parameters do not differ significantly from one model to another. However, the careful analysis of these values shows that the Langmuir model is the most suitable for describing the experimental data, suggesting that the adsorption of RIF and RIX on PET fibers occurs in a single layer. The maximum adsorption capacity (q_max_, mg/g) is almost 3 times higher in the case of RIF than in the case of RIX (Table 3), which demonstrates that RIF has a stronger tendency to bind to the surface of PET fibers compared to RIX. Moreover, in both the case of RIF and RIX, the maximum adsorption capacity values calculated from the Langmuir model (44.84 mg RIF/g and 15.65 mg RIX/g) (Table 3) are fairly close to those obtained experimentally (32.06 mg RIF/g and 14.23 mg RIX/g), which explains the flattening of the experimental adsorption isotherms (Figure 7) at high concentrations of the two antibiotics. Most likely, the adsorption of RIF and RIX occurs only on the surface of PET fibers (in accordance with the Langmuir model), which involve physicochemical interactions between antibiotics and active sites of the adsorbent (according to the values of parameter *B* from the Temkin model) (Table 3); this behavior makes adsorption processes favorable events at high concentrations of antibiotics (according to the values of parameter *n* from the Freundlich model) (Table 3).

The maximum adsorption capacity values obtained for the retention of RIF and RIX on PET fibers are comparable to those reported in the literature for various adsorbents under similar experimental conditions. Several examples are presented in Table 4.

All these aspects highlight the applicative potential of PET fibers in the removal of RIF and RIX from aqueous environments and encourage further research.

**Figure 8:**
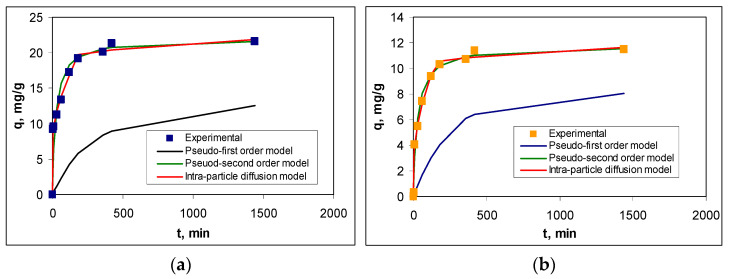
Experimental and calculated kinetic curves for adsorption of RIF (**a**) and RIX (**b**) onto PET fibers (experimental conditions: pH = 2.0, adsorbent dose = 0.4 g/L, initial concentration = 21.1 mg/L, and temperature = 22 ± 1 °C).

Figure 8 illustrates the experimental kinetic curves and those obtained using the pseudo-first order, pseudo-second order, and intra-particle diffusion kinetic models, while the kinetic parameters are summarized in Table 5. And in this case, the selection of the most suitable model was made using statistical parameters (R^2^, RMSD, and Chi-square).

The statistical parameters presented in Table 5 show that the adsorption of RIF and RIX on PET fibers is best described by the pseudo-second order kinetic model. The agreement between the experimental data and this kinetic model is also supported by the calculated equilibrium adsorption capacity values (q_e,cal_, mg/g), which are close to those obtained experimentally (q_e,exp_, mg/g) for both antibiotics (Table 5). This suggests that the adsorption processes of RIF and RIX on PET take place through physicochemical interactions between the functional groups of antibiotics and two active centers on the surface of the PET fibers.

The necessity for RIF and RIX molecules to interact with two functional groups (active centers) on the surface of PET fibers results in (i) a high initial adsorption rate when most functional groups on the adsorbent surface are free, followed by a decrease in this rate as the superficial functional groups become occupied, and the adsorption process reaching equilibrium in a short time (maximum 180 min); and (ii) a different adsorption efficiency for RIF and RIX under the same experimental conditions (Figure 8), indicating the differing availability of functional groups in the two antibiotics to participate in such interactions, a factor closely linked to their chemical structure. However, as can be seen from Table 5, the rate constants obtained for the pseudo-second order kinetic model (k_2_, g/mg min) have the same order of magnitude, which demonstrates that the same types of interactions occur during the adsorption processes for both antibiotics.

Although the pseudo-first order kinetic model leads to adsorption capacity values (q_e,cal_, mg/g) that differ significantly from those obtained experimentally (q_e,exp_, mg/g), the regression coefficient (R^2^) and the rate constants (k_1_, 1/min) have comparable values to those obtained for the pseudo-second order model (Table 5). These comparable values allow us to state that, most likely, the adsorption of RIF and RIX on PET fibers occurs in two stages: First, the antibiotic molecules interact with a single functional group (following pseudo-first order kinetics), after which the second interaction takes place and the kinetics transition to pseudo-second order. The second interaction serves to immobilize RIF and RIX on the surface of PET fibers, thus creating a second binding site for the large antibiotic molecules.

The contribution of elementary diffusion processes to the adsorption of RIF and RIX on PET fibers is highlighted by the intra-particle diffusion model (Figure 8). According to this model, diffusion processes are not rate-determining steps (the linear dependency of q vs. t^1^/^2^ does not pass through the origin), and two regions can be identified (Figure S1): Region I, which corresponds to the diffusion of RIF and RIX from the bulk solution to the surface of PET fibers, and Region II, which corresponds to the diffusion of antibiotics into the pores of PET fibers [47,48]. Moreover, the values of the kinetic parameters calculated for this model (Table 5) show that the rate constants corresponding to Region I are an order of magnitude higher than those corresponding to Region II, while the antibiotic concentration (c, mg/L) is higher in Region II than in Region I. These differences suggest that the diffusion of antibiotic molecules from the bulk solution to the surface of the PET fibers occurs much more easily compared to their diffusion into the pores of the adsorbent. This is mainly due to the smooth surface of the PET fibers (as demonstrated by the SEM images—Figure 3b), which allows RIF and RIX to bind only to the surface of the adsorbent (in accordance with the Langmuir model—Table 3). It should also be noted that, compared to RIX, in the case of RIF, the elementary diffusion processes are faster, which results in both the rate constants and the c values (mg/L) being higher for both regions (Table 5). These variations are most likely determined by the chemical structure of the two antibiotics. Although RIF has a higher molecular weight (822.405 g/mol) compared to RIX (782.879 g/mol), the presence of an additional heterocycle in the RIX molecule (Table 1) alters the mobility of these molecules. Therefore, RIF molecules move more easily through the aqueous solution than RIX, and this influences the efficiency of the adsorption processes.

### 3.4. Thermodynamic Parameters

Experiments on the influence of temperature (Figure 4d) showed that increasing the temperature from 10 to 50 °C causes an increase in the adsorption capacity by 74.27% in the case of RIF and 43.67% in the case of RIX. This behavior suggests that, regardless of the nature of the antibiotic, the adsorption process onto PET fibers is heat-absorbing. To obtain a quantitative evaluation of the thermodynamic behavior of RIF and RIX adsorption onto PET fibers, the thermodynamic parameters (∆G^0^, ∆H^0^, and ∆S^0^) were calculated using the van’t Hoff equations [49]. The values obtained for these parameters are presented in Table 6, while the linear representations required for the calculation of ∆H^0^ are illustrated in Figure S2.

As can be seen from Table 6, the Gibbs free energy values (ΔG^0^) are negative for all temperature values, both for RIF and RIX, which indicates that the adsorption of the two antibiotics onto PET fibers is a spontaneous process. Moreover, the ΔG^0^ values do not differ significantly depending on the nature of the antibiotic, which suggests that the adsorption of RIF and RIX onto PET fibers occurs through a similar mechanism. The enthalpy variation values (ΔH^0^) are positive (Table 6), which indicates that the adsorption processes of RIF and RIX onto PET fibers are endothermic and are favored by increasing temperature. This observation is consistent with the experimental results (Figure 4d), which showed that raising the temperature from 10 to 50 °C leads to an increase in the adsorption capacity for both RIF and RIX. However, two observations must be made, namely (i) low values of this parameter indicate that the binding of antibiotic molecules to the surface of PET fibers does not involve breaking or forming chemical bonds, most likely, the interactions that occur in the adsorption processes are physicochemical (electrostatic, hydrogen bonds, π-π interactions), and (ii) the difference of one order of magnitude between the ΔH^0^ value in the case of RIF compared to that obtained for RIX (Table 6) suggests that in the adsorption of RIX, the share of physical interactions (such as hydrogen bonds or π-π interactions) is greater than in the case of RIF. This hypothesis is supported by the experimental results presented in the previous sections, which clearly highlight the higher efficiency of RIF adsorption on PET fibers compared to RIX. The positive values of the entropy variation (ΔS^0^) (Table 6) indicate an increase in the degree of disorder at the adsorbent–aqueous solution interface during the adsorption processes. This increase in disorder is most likely due to the water molecules that hydrate the antibiotic molecules, which are released as RIF and RIX bind to the adsorbent surface. The similar values of this parameter obtained for the two antibiotics suggest that their adsorption onto the surface of PET fibers occurs through a similar mechanism, involving the same type of interactions. All these observations show that the adsorption of RIF and RIX onto PET fibers is a spontaneous, endothermic, and thermodynamically feasible process, which may have practical applications.

### 3.5. Effect of Co-Existing Ions

As was shown in the previous section (Section 3.1), in a strongly acidic environment (pH = 2), established as optimal for these adsorption processes, the RIF and RIX molecules are negatively charged (due to the ionization of the first functional group), while the functional groups of the PET fibers are undissociated/partially protonated. Under these conditions, anions are expected to have a more pronounced influence on the efficiency of the adsorption processes than cations; this is the reason why only anions were selected for the experimental studies.

**Figure 9:**
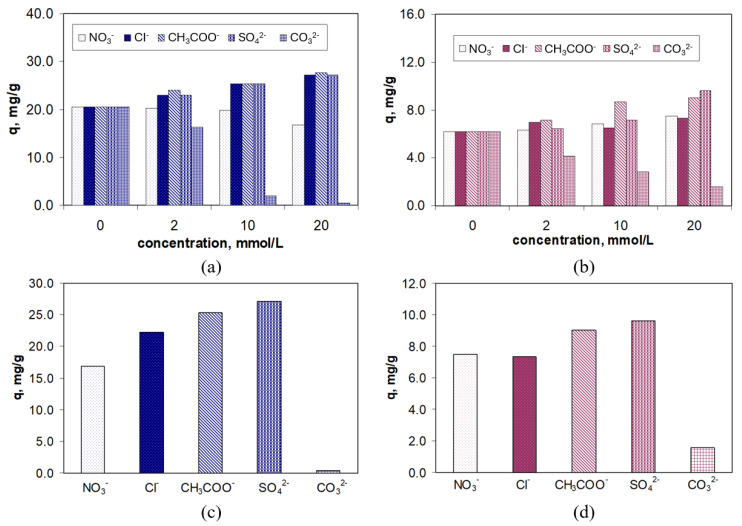
Effect of co-existing anions on adsorption of RIF (**a**,**c**) and RIX (**b**,**d**) onto PET fibers (experimental conditions: pH = 2.0, adsorbent dose = 0.4 g/L, initial concentration = 21.1 mg/L, and temperature = 22 ± 1 °C, in the case of Figure 9c,d, concentration of anions = 20 mmol/L).

The experiments were carried out under optimal conditions (pH = 2.0; adsorbent dose = 0.4 g/L, contact time = 180 min, temperature = 22 ± 1 °C), for an initial antibiotic concentration of 21.1 mg/L, while the anion concentration (Cl^−^, NO_3_^−^, CH_3_COO^−^, SO_4_^2−^, and CO_3_^2−^) varied between 2.0 and 20.0 mmol/L. The results obtained are illustrated in Figure 9.

As can be seen in Figure 9, the presence of inorganic anions in the aqueous solution leads to an increase in the adsorption capacity for both RIF and RIX, with this increase becoming more pronounced as the concentration of anions rises. Moreover, the increase in adsorption capacity is more pronounced for RIF (maximum 7.6 mg/g) than for RIX (maximum 3.6 mg/g) (Figure 9a,b), but regardless of the type of antibiotic, it follows the order of NO_3_^−^ < Cl^−^ < CH_3_COO^−^ < SO_4_^2−^ (Figure 9c,d). This order corresponds to the Hofmeister series, which orders anions based on their hydration energy [50]. Thus, anions with high hydration energies (e.g., SO_4_^2−^; ∆H^0^_hydr._ = −1025 kJ/mol) lead to a greater increase in the adsorption capacity of RIF and RIX on PET fibers compared to anions with low hydration energies (e.g., NO_3_^−^, ∆H^0^_hydr._ = −380 kJ/mol) (Figure 9c,d).

Based on these experimental results, it can be said that the presence of anions leads in the ordering of water molecules in the aqueous solution, an effect that becomes more significant as the hydration energy of the anion increases. As a result, the number of free water molecules or those hydrating RIF and RIX decreases, and consequently, the antibiotic molecules are “pushed” toward the surface of the PET fibers (which are also slightly hydrated). This “pushing” effect of antibiotic molecules toward the surface of the PET fibers also depends on their water solubility (Table 1), and is more pronounced in the case of RIF (which is more readily soluble in water, thus binding a larger number of water molecules upon dissolution) compared to RIX (which is less soluble in water and therefore the number of water molecules bound upon dissolution is lower) (Figure 9c,d). However, even the presence of high concentrations of inorganic salts does not significantly improve the removal percentages of RIF and RIX on PET fibers, with the increase in this parameter being a maximum of 14% for RIF and 6% for RIX.

An exception is the CO_3_^2−^ ion. As shown in Figure 9a,b, the presence of the CO_3_^2−^ ion leads to a decrease in the adsorption capacity for both RIF and RIX, and this decrease becomes more significant as the concentration of the CO_3_^2−^ ion increases. This behavior may be due to (i) the strongly acidic media used in the experimental studies (pH = 2.0), which changes the speciation form of the carbonate ions, or (ii) the direct interaction between the superficial functional groups of PET fibers and the carbonate ions, which prevents the adsorption of RIF and RIX. The first assumption is rather unlikely. Although at pH = 2.0 the predominant speciation form is CO_2_ (according to the speciation diagram [51]), no gas bubble formation indicating CO_2_ release was observed experimentally. It is much more likely that the CO_3_^2−^ ions in the aqueous solution interact directly with the functional groups on the surface of the PET fibers (due to structural similarities [52,53]), thereby blocking some functional groups of the adsorbent, which can no longer be involved in the adsorption process. The possibility of such interactions has already been reported in the literature for various materials with functional groups containing O-donor atoms in the presence of carbonate ions [52,53].

All these observations provide a more comprehensive understanding of the adsorption process of RIF and RIX onto PET fibers and are valuable both for the formulation of an adsorption mechanism and for practical applications.

### 3.6. Mechanism of RIF and RIX Adsorption on PET Fibers

The experimental results presented in the previous sections showed that (i) the adsorption of RIF and RIX onto PET fibers occurs with maximum efficiency when pH = 2.0, adsorbent dose = 0.4 g/L, contact time = 180 min, and temperature = 22 °C (see Section 3.1); (ii) the adsorption takes place in a monolayer, is described by the Langmuir model, and involves the interaction between RIF and RIX with two functional groups on the adsorbent surface (see Section 3.3); and (iii) the adsorption processes are spontaneous and endothermic (see Section 3.4).

**Figure 10:**
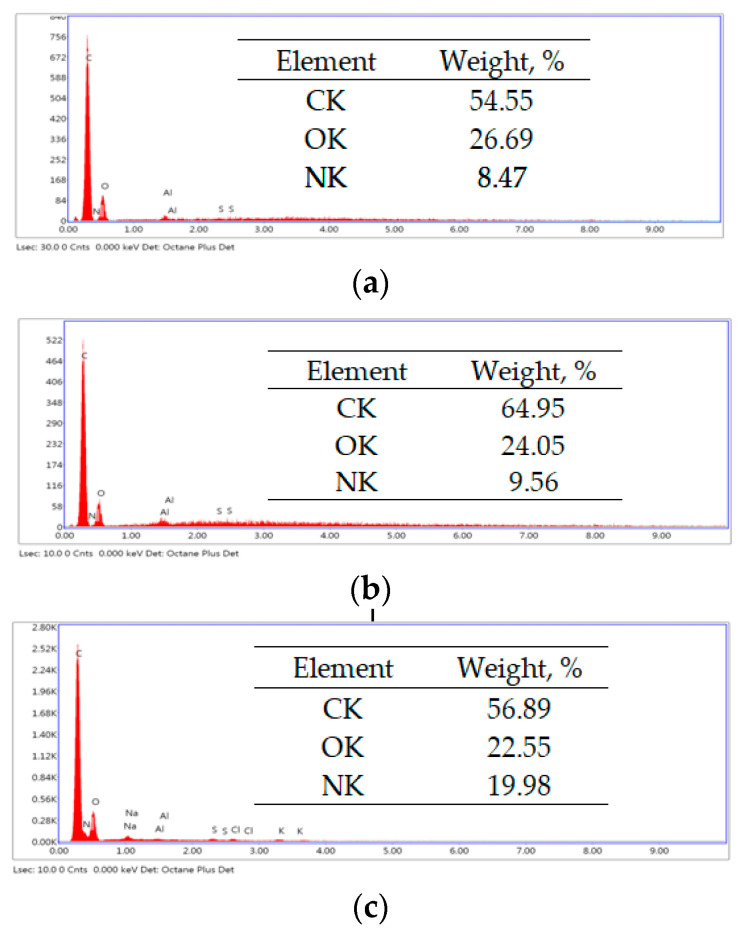
EDX spectra of PET fibers before (**a**) and after adsorption of RIF (**b**) and RIX (**c**).

At this pH value, RIF and RIX are negatively charged due to the dissociation of hydroxyl-type groups (pKa_1_ < 2.0) (Table 1), while the functional groups of the adsorbent are protonated (positively charged). These conditions promote favorable electrostatic interactions between RIF, RIX, and the adsorbent surface. Consequently, the conditions are favorable for facilitating electrostatic interactions between the two antibiotics and the adsorbent. The spontaneous retention of RIF and RIX on PET fibers is demonstrated by the EDX spectra (Figure 10).

**Figure 11:**
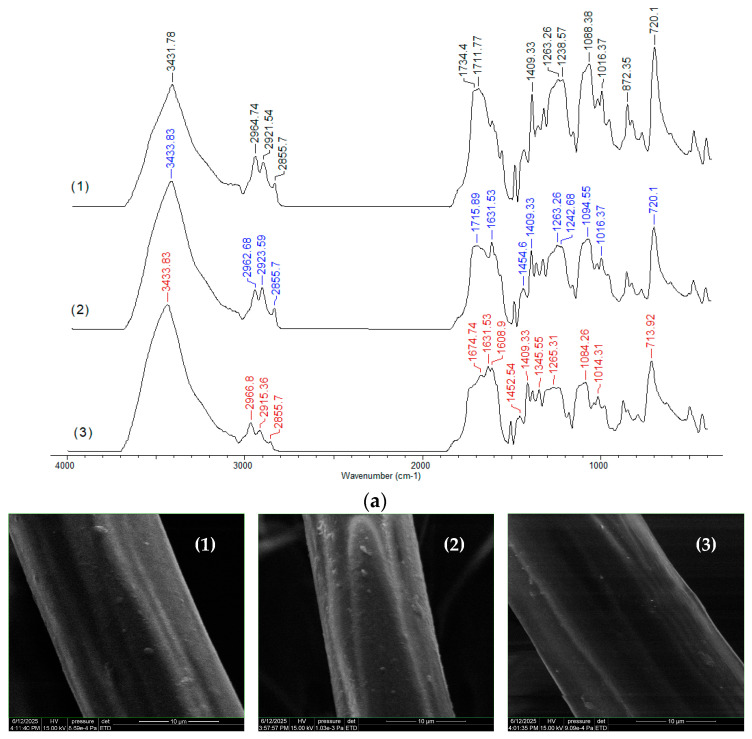

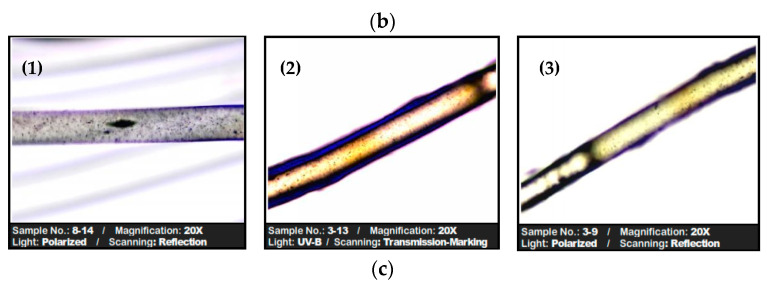
FTIR spectra (**a**) and SEM (**b**) and optical (**c**) images of PET fibers before (1) and after adsorption of RIF (2) and RIX (3).

The increase in carbon and nitrogen content after the adsorption of RIF and RIX (as shown in Figure 10) provides evidence that both antibiotics are retained on the surface of the PET fibers. Furthermore, the “binding” of antibiotic molecules to the functional groups of the adsorbent predominantly through electrostatic interactions is evidenced by the FTIR spectra (Figure 11a).

Comparison of the FTIR spectra of PET fibers before and after the adsorption of RIF and RIX (Figure 11a) reveals that (i) no additional new bands appear in the spectra, indicating that the formation of highly covalent bonds is negligible; and (ii) the most significant shifts in absorption maxima correspond to C=O (1734–1711 cm^−1^ in spectrum 1→1715–1631 cm^−1^ and 1674–1631 cm^−1^ in spectra 2 and 3) and C–O–C bonds (1088 cm^−1^ in spectrum 1→1094 cm^−1^ and 1084 cm^−1^ in spectra 2 and 3), suggesting that these functional groups are most likely involved in the adsorption processes.

The changes in the maxima of the other absorption bands in the FTIR spectra are insignificant (within the range of ±4–6 cm^−1^, determined by the resolution of the spectral recordings), which shows that the adsorption of RIF and RIX onto the PET fibers does not even alter the chemical proximity of the functional groups on the surface. This observation is also supported by microscopic images (SEM and optical) (Figure 11b,c), where it can be seen that the structure of the PET fibers is not affected after antibiotic adsorption, but they only “cover” the surface of the adsorbent.

**Figure 12:**
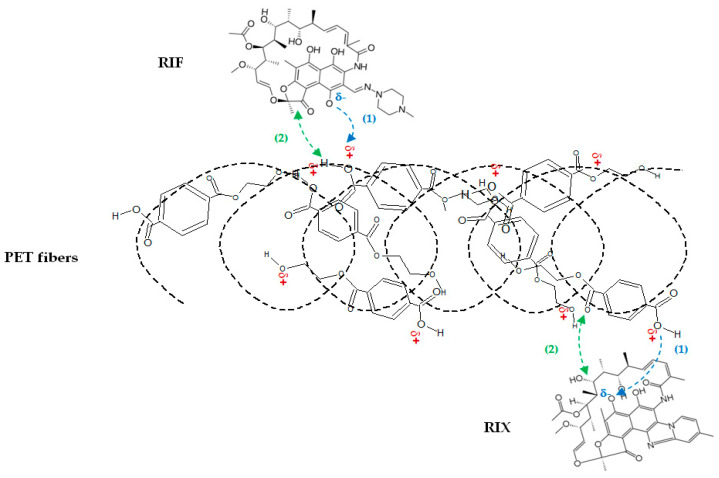
Schematic illustration of adsorption mechanism of RIF and RIX antibiotics on PET fibers.

Taking all these aspects into account, it can be said that the adsorption of RIF and RIX onto PET fibers involves two stages (Figure 12), namely (1) electrostatic interactions between the ionized functional groups of RIF and RIX and the superficial functional groups of the adsorbent (these occur rapidly and explain the need for a strongly acidic media (pH = 2) and the spontaneity of the adsorption processes), followed by (2) the “stabilization” of the antibiotic molecule on the surface of the PET fibers through physical interactions such as hydrogen bonding or π–π interactions (these require a longer period of time (180 min) and explain the correspondence between the experimental data and the Langmuir and pseudo-second order models).

The retention of RIF and RIX on PET fibers through these types of interactions has particularly important practical implications. One of the most significant is that desorption of the antibiotics can be achieved even by treatment with inorganic salt solutions (strong electrolytes), which are capable of breaking the electrostatic bonds formed during the adsorption processes. Our preliminary studies have shown that when PET fibers loaded with RIF or RIX are treated with a medical-grade polyelectrolyte solution (hydration salt solution, purchased from Biomega Natural Nutrients S.L., Madrid, Spain), desorption percent can reach approximately 40% in 30 min of contact time. However, the selection of the most suitable desorption agent, but especially its solution volume and concentration, requires much more rigorous experiments that must be discussed in detail. Therefore, these results will be presented in a subsequent study.

## 4. Conclusions

This study examined the adsorption of rifampicin (RIF) and rifaximin (RIX) antibiotics from aqueous media on PET (polyethylene terephthalate) fibers, obtained by mechanically processing PET waste. The experiments were performed at different pH values, adsorbent dose, contact time, initial antibiotic concentration, and temperature; the experimental values of these parameters were analyzed using a neuro-evolutive technique (ANE) combining sequential deep learning (DL) models with a differential evolution algorithm to establish the optimal adsorption conditions. The highest adsorption capacity values were obtained at pH = 2, adsorbent dose = 0.4 g/L, contact time = 180 min, and temperature = 22 °C. Also, the optimal ANN-DL algorithm was used to adequately describe the adsorption dynamics of RIF and RIX on PET fibers. Modeling isotherms and kinetic data showed that the adsorption processes of RIF and RIX on PET fibers are spontaneous and endothermic (ΔG < 0, ΔH > 0), and are described by the Langmuir model (R^2^ > 0.97) and the pseudo-second order kinetic model (R^2^ > 0.99). In addition, FTIR spectra and microscopic images revealed that the adsorption of antibiotics on PET fibers most likely involves physicochemical interactions. The presence of inorganic anions in the aqueous solution leads to a moderate increase in the adsorption capacities of RIF (max. 7.6 mg/g) and RIX (max. 3.6 mg/g). Based on the experimental results presented in this study, an adsorption mechanism of RIF and RIX on PET fibers was developed, which is particularly useful for the potential practical applications of these adsorption processes.

## Figures and Tables

**Table 1:** Chemical characteristics of RIF and RIX antibiotics.

Characteristic	RIF	RIX	Reference
Chemical formula	C_43_H_58_N_4_O_12_	C_43_H_51_N_3_O_11_	[36,37]
Chemical structure	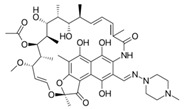	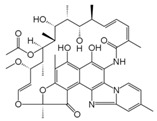	
Molecular mass, g/mol	822.405	785.352	
Dissociation constant (pK_a_)	1.8; 7.9	2.08; 3.02; 7.28; 11.32; 12.55	[37,38]
Solubility, mg/L	50.0	12.0	

**Table 2:** Statistical indicators for best model.

Sub-Set	Indicator	Value
Training	R^2^	0.961
	MAE	2.994
	MSE	21.016
	MAPE	0.182
Testing	R^2^	0.655
	MAE	7.266
	MSE	141.239
	MAPE	0.484

R^2^ = coefficient of determination; MAE = mean absolute error; MSE = mean squared error; MAPE = mean absolute percentage error.

**Table 3:** Isotherm parameters for adsorption of RIF and RIX onto PET fibers.

Model	Equation	Parameter	RIF	RIX
Langmuir	$q=\frac{q_{\max}\cdot K_{L}\cdot c}{1+K_{L}\cdot c}$	R^2^	0.9871	0.9791
		RMSD	0.024	0.043
		Chi-square	0.016	0.022
		q_max_, mg/g	44.84	15.63
		K_L_, L/g	0.0731	0.1141
Freundlich	$q=K_{F}\cdot c^{1/n}$	R^2^	0.8799	0.9793
		RMSD	0.034	0.076
		Chi-square	0.029	0.086
		1/n	0.43	0.23
		K_F_, L/g	6.6054	5.1642
Temkin	$q=B\cdot \ln(A_{T}\cdot c)$	R^2^	0.9461	0.9209
		RMSD	0.019	0.083
		Chi-square	0.012	0.078
		A_T_, L/g	8.55	2.08
		B, J/mol	810.53	893.79

*q* is the adsorption capacity; *q_max_* is the maximum adsorption capacity; *K_L_* is the Langmuir constant; *c* is the equilibrium concentration of RIF and RIX; *K_F_* is the Freundlich constant; *n* is the heterogeneity factor; *A_T_* is the equilibrium binding constant; and *B* is the constant correlated with the heat of adsorption.

**Table 4:** Maximum adsorption capacities obtained for adsorption of RIF and RIX on various adsorbents.

Adsorbent	q_max_, mg/g		Reference
	RIF	RIX	
Alkali-activated kaolin	8.29	-	[42]
Calcined *Mytella falcata* shells	20.70	-	[43]
Acid-activated Iraqi red mud	195.69	-	[44]
Activated carbon with ZnCl_2_	476.2	-	[45]
NiFe_2_O_4_/GO nanocomposite	-	30.12	[46]
PET fibers	44.84	15.63	This study

**Table 5:** Kinetic parameters for adsorption of RIF and RIX onto PET fibers.

Model	Equation	Parameter		RIF	RIX
		q_e,exp_, mg/g		21.58	11.51
Pseudo-first order model	$q_{t}=q_{e}\cdot (1-e^{-k_{1}\cdot t})$	R^2^		0.9312	0.9089
		RMSD		0.117	0.051
		Chi-square		0.823	1.204
		q_e,cal_, mg/g		12.64	8.07
		k_1_, 1/min		0.0034	0.0039
Pseudo-second order model	$q_{t}=\frac{k_{2}\cdot q_{e}^{2}\cdot t}{1+k_{2}\cdot q_{e}\cdot t}$	R^2^		0.9994	0.9997
		RMSD		0.024	0.007
		Chi-square		0.096	0.016
		q_e,cal_, mg/g		21.93	11.75
		k_2_, g/mg min		0.0019	0.0031
Intra-particle diffusion model	$q_{t}=k_{diff}\cdot t^{1/2}+c$	I	RMSD	0.052	0.011
			Chi-square	0.002	0.028
			R^2^	0.9799	0.9957
			k_diff_, mg/g min^1/2^	0.9238	0.6985
			c, mg/L	6.67	1.81
		II	R^2^	0.6462	0.6253
			k_diff_, mg/g min^1/2^	0.0846	0.0432
			c, mg/L	18.62	9.99

where *q_e_*, *q_t_* are the adsorption capacity at equilibrium and at different times; *k*_1_ is the rate constant of the pseudo-first order model; *k*_2_ is the rate constant of the pseudo-second order model; *k_diff_* is the intra-particle diffusion rate constant; *c* is the concentration of RIF and RIX; I and II are the linear regions obtained for the intra-particle diffusion model.

**Table 6:** Thermodynamic parameters for adsorption of RIF and RIX onto PET fibers.

Antibiotic	T, K	∆G^0^, kJ/mol	∆H^0^, kJ/mol	∆S^0^, J/mol K
		∆G^0^ = −RT lnK_L_	${\mathrm{lnK}}_{L}=\frac{\Delta H^{0}}{RT}+\frac{\Delta S^{0}}{R}$	∆S^0^ = (∆H^0^ − ∆G^0^)/T
RIF	283.15	−13.07	0.41	47.61
	293.15	−10.26		36.38
	323.15	−10.30		33.12
RIX	283.15	−12.41	0.07	43.81
	293.15	−12.40		42.28
	323.15	−13.43		41.57

∆G^0^ is the variation in free Gibbs energy; ∆H^0^ is the variation in enthalpy; ∆S^0^ is the variation in entropy; R is the universal gas constant (8.314 J/K mol); T is the temperature (K); K_L_ is the Langmuir constant (L/g), calculated for each temperature.

## Data Availability

The original contributions presented in the study are included in the article; further inquiries can be directed to the corresponding author.

## Supplementary Materials

The following supporting information can be downloaded at: https://www.mdpi.com/article/10.3390/polym17152089/s1. Figure S1: Linear representations of intra-particle diffusion model for adsorption of RIF (a) and RIX (b) on PET fibers; Figure S2: Dependencies: ln K_L_ vs. 1/T.
