# Supplementary material for: Removal of Rifampicin and Rifaximin Antibiotics on PET Fibers: Optimization, Modeling, and Mechanism Insight
Source: Polymers (Basel). 2025 Jul 30;17(15):2089. doi: 10.3390/polym17152089 (PMC12349532; doi:10.3390/polym17152089)

Figure S1: Linear representations of the intra-particle diffusion model for the adsorption of RIF (a) and RIX (b) on PET fibers.

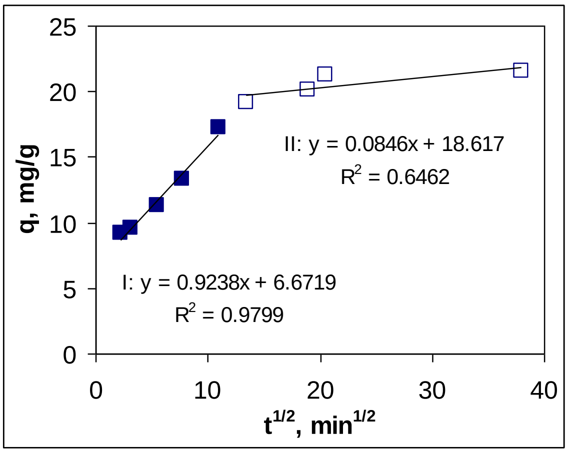

(a)

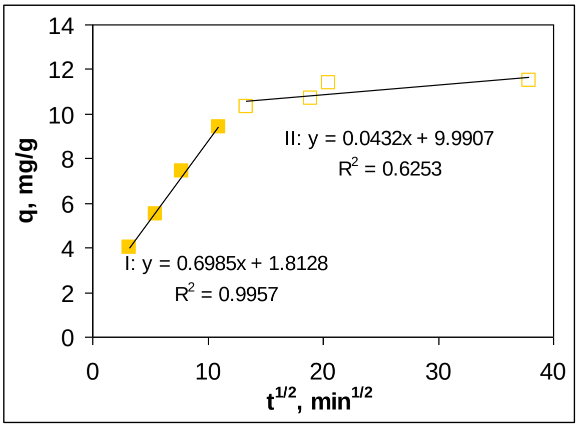

(b)

Figure S2: Dependencies: ln K_L vs. 1/T.

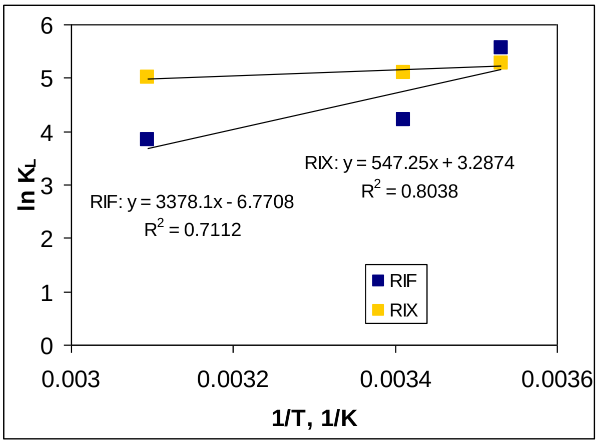

Supplement: Supplementary file 1 [file polymers-17-02089-s001.zip › polymers-3757907-supplementary.pdf]
